# Marginal Entropion: A Frequently Overlooked Eyelid Malposition

**DOI:** 10.4274/tjo.20591

**Published:** 2015-10-05

**Authors:** Mustafa Erdoğan, Şeyda Karadeniz Uğurlu

**Affiliations:** 1 Gaziemir Nevvar Salih İşgören Government Hospital, Clinic of Ophthalmology, İzmir, Turkey; 2 Katip Çelebi University Faculty of Medicine, Department of Ophthalmology, İzmir, Turkey

**Keywords:** Entropion, trichiasis, meibomianitis, eyelid surgery

## Abstract

**Objectives:**

To evaluate the clinical findings and outcomes of surgical treatment in patients with marginal entropion.

**Materials and Methods:**

Patients with impairment of the natural square-shaped eyelid margin morphology, anterior migration of mucocutaneous junction and mild lid inversion toward the ocular surface were diagnosed as having marginal entropion. Patients with shortened fornices, cicatricial changes or subconjunctival fibrosis were excluded. Demographic characteristics, ophthalmologic examination findings, surgical procedures and follow-up data were evaluated retrospectively.

**Results:**

Twelve eyes of 11 patients were included in the study. Median age was 73 years (range, 49-84 years). All cases presented with signs of meibomianitis and were treated preoperatively with oral doxycycline and topical corticosteroids. Tarsal fracture procedure was performed for correction of lid malposition. In all patients, lid malposition was corrected and ocular irritation findings had regressed. No recurrences were observed in the follow-up period of mean 10 months (range, 5-16 months).

**Conclusion:**

Marginal entropion is a common malposition that is frequently misdiagnosed as trichiasis and is overlooked. Complications secondary to misdiagnosis can be avoided and a normal lid position achieved when the correct diagnosis is made.

## INTRODUCTION

Entropion, or eyelid inversion, may develop as a result of congenital, involutional or cicatricial causes. The underlying cause of cicatricial entropion is chronic tarsoconjunctival inflammation and cicatrization.^1^ Various clinical manifestations of cicatricial changes can be seen in proportion to the size of the affected area. In chemical and physical injuries and autoimmune diseases like cicatricial pemphigoid, prominent findings may include macroscopic scar tissue, symblepharon and fornix obliteration. These conditions cause complaints in the early stages, and so are easily diagnosed. However, in patients with chronic blepharitis, mild cicatricial changes affecting the eyelid margins may be overlooked.

Marginal entropion is a mild form of cicatricial entropion associated with chronic eyelid inflammation.^2,3^ In this presentation, in addition to eyelid inversion, the posterior edge of the eyelid adjacent to the globe loses its squared edges and becomes rounded. Anterior migration of the mucocutaneous junction also indicates a diagnosis of marginal entropion.^2^

Trichiasis is misdirection of the eyelashes toward the globe, and can occur independently or together with marginal entropion. Marginal entropion may be mistaken for trichiasis in the clinic. Nerad2 pointed out the need for distinction between marginal entropion of the lower eyelid and trichiasis. Most patients diagnosed with trichiasis are found to have marginal entropion.^2,4^ The differentiation of this entropion subtype from trichiasis and other entropion cases is important for treatment planning.

There are very few studies in the literature on this topic.^5,6,7^ The current study examined treatment methods and subsequent outcomes in consecutive patients with marginal entropion, and emphasized the importance of differential diagnosis of this condition.

## MATERIALS AND METHODS

The study included 11 patients (12 eyes) who presented to the Division of Oculoplastic Surgery, İzmir Katip Çelebi University Atatürk Training and Research Hospital between January 2010 and July 2011, were diagnosed with marginal entropion and underwent surgical treatment. Informed consent was obtained from all patients. Patients’ demographic data, examination findings, surgical procedures and follow-up results were evaluated retrospectively. Marginal entropion was diagnosed when impairment of the natural square-shaped eyelid margin morphology, anterior migration of mucocutaneous junction and mild lid inversion toward the ocular surface were observed.^2,3^ Patients with shortened fornices, cicatricial changes or subconjunctival fibrosis were categorized as cicatricial entropion and excluded. Cases with accompanying trichiasis and distichiasis and patients who had previously undergone conjunctival or eyelid surgery were not included in the study.

**Figure 1:**
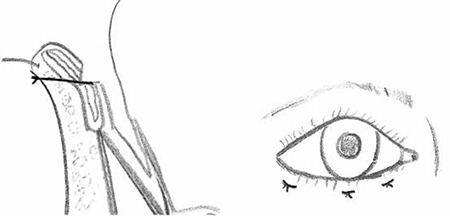
Lower eyelid tarsal fracture surgery consisted of a tarsal incision that left the anterior lamella intact and the placement of three everting sutures

To suppress chronic meibomianitis, all patients were treated preoperatively for at least 1 month with 100 mg oral doxycycline (Monodoks®, Deva, Turkey) once daily, topical 0.1% fluorometholone (FML®, Allergan) four times daily as an anti-inflammatory therapy, and lubricating tear drops. Following this medical treatment, tarsal fracture procedure was performed under local anesthesia. A full-layer transverse incision was made to the lower tarsal plate approximately 2 mm from the edge of the eyelid, leaving the anterior lamellae intact. In cases in which entropion did not span the entire eyelid, the tarsal incision was restricted to the area of entropion. Three everting sutures were placed using 6-0 polyglactin (Vicryl; Ethicon, Inc.) (Figure 1). The patients’ postoperative results were recorded. Eyelid position and changes in patient symptoms were evaluated.

## RESULTS

**Figure 2:**
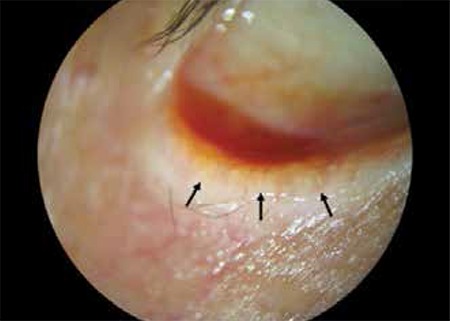
A patient with eyelash loss due to chronic blepharitis. The mucocutaneous junction has anteriorized beyond the meibomian gland orifices, and has reached the base of the eyelash follicles. The arrows indicate the mucocutaneous junction (Lower eyelid has been manually inverted to provide a better image of the mucocutaneous junction)

**Figure 3:**
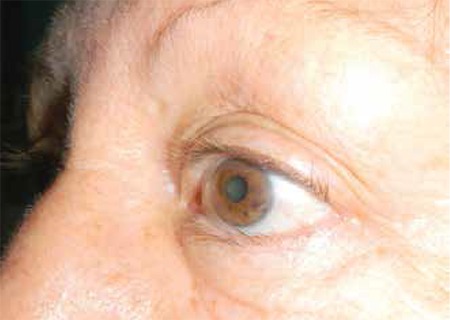
Preoperative appearance of patient with marginal entropion

The patients had a median age of 73 years (range, 49-84 years); there were 5 men and 6 women. All patients had symptoms related to ocular irritation and presented with chronic meibomianitis. Median duration of symptoms of ocular irritation was 12 months (range, 6-60 months). Characteristically, there was loss of the natural square-shaped eyelid margin morphology; all patients displayed rounding of the posterior eyelid edge and anterior migration of the mucocutaneous junction (Figures 2 and 3). In one case, entropion was accompanied by a mass located distant from the free edge of the lower lid. In this patient, an incision was made to remove the mass in addition to the tarsal incision for eyelid malposition.

**Figure 4:**
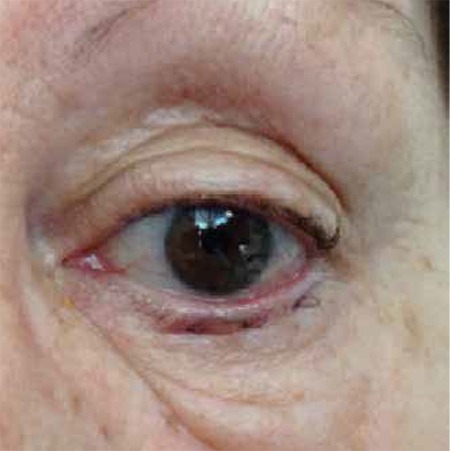
Postoperative week 1 appearance of the lower lid of the patient from Figure 3. The lashes have taken their normal curvature and are directed away from the globe, while the lower lid has assumed its ideal position over the globe

All ocular symptoms disappeared after one week. All patients had ideal eyelid position; eyelashes no longer touched the globe (Figure 4). There were no recurrences of eyelid malposition during the follow-up period (10.1±4.4 months).

## DISCUSSION

A thorough examination is crucial for the proper diagnosis and treatment of patients presenting with irritation resulting from contact between the lower eyelashes and the globe. Eyelash contact with the globe may be caused by trichiasis, metaplastic lashes, distichiasis or entropion. As these conditions may present together or alone, differential diagnosis is essential for the determination of appropriate treatment. Marginal entropion describes a situation in which there is not significant inversion of the eyelid, but the mucocutaneous junction advances anteriorally to the meibomian gland orifices, which results in the misdirection of the lashes. This condition may be directly perceived as a pathology of the lashes instead of its correct designation as eyelid malposition. However, with a detailed anterior segment examination, marginal entropion can often be easily distinguished from trichiasis. In marginal entropion, slight inversion of the eyelid margin, conjunctivalization of the area surrounding the meibomian gland orifices and the anterior migration of the mucocutaneous junction can be observed. The stratified squamous epithelium that contains the meibomian gland orifices contrasts with the conjunctival epithelium, making diagnosis easier.^3,8^

The anterior migration of the mucocutaneous junction was first described as an early finding of upper lid entropion in a study by Jones et al.^9^ It was shown through electron microscopic analysis that the mucocutaneous junction is located between the lashes and the meibomian gland orifices and the transition area created an exclusive demarcation line. It is believed that the tear film layer is influential in the metaplastic changes seen in the lid margin.^10^ Keratinization posterior to the meibomian gland orifices in ectropion supports this view.

There are a limited number of studies in the literature about marginal entropion. Searching for the key words ‘marginal entropion and lower eyelid’ in PubMed yields only one study which analyzed cases diagnosed with marginal entropion of the lower eyelid.^5^ Many cases of cicatricial entropion treated with the tarsal fracture procedure appear in the literature.^11,12,13,14,15,16,17^ These patient populations, which also include cases of marginal entropion, consist largely of patients with trachoma-related upper lid entropion. Barber and Dabbs10 showed that among 116 patients diagnosed with trichiasis, 95 (82%) had eyelid margin abnormalities. In these patients, the most frequently seen form of eyelid margin abnormality (in 69%) was anterior migration of the mucocutaneous junction together with “a small degree of entropion”. This study reveals that patients diagnosed as having trichiasis with “a small degree of entropion” are frequently marginal entropion cases.

Choi et al.^5^ analyzed 30 lower eyelids of 22 patients diagnosed with marginal entropion and described the diagnostic criteria as anterior migration of the mucocutaneous junction, lack of severe cicatrization of the conjunctiva and contact between the eyelashes and the globe. With an average age of 58 years, that study included younger patients than our study population. In our study there were no factors other than meibomianitis, whereas there was history of long-term glaucoma medication use in 4 eyelids and suspected trauma in 3 eyelids of Choi et al.’s^5^ patient group. Furthermore, while 90% of the patients in that study underwent epilation and/or laser ablation, electrolysis and eyelid surgery, the patients in our study only underwent epilation preoperatively.

Treatment of marginal entropion should be planned according to the etiopathogenesis. The mechanical removal of the eyelashes or attempts at follicle elimination have been described.^18,19^ However, the aggressive use of laser epilation, cryotherapy or electrolysis can increase scarring, and may turn marginal entropion into a more pronounced form of entropion.^5^ Even following complete eyelash ablation, contact of the eyelid, sebum and sweat with the eye can cause continued irritation. For these reasons, patients with possible marginal entropion should be identified and aggressive trichoablation should be avoided.

There are many surgical techniques described in the literature for the correction of cicatricial entropion.^4,7,20,21,22,23^ Among these techniques are eyelash resection, anterior lamellar recession, posterior lamellar advancement, eyelid rotation with full-thickness blepharotomy, mucous membrane grafting and tarsal fracture operation. Successful results have been reported by applying these methods to treat cicatricial entropion arising from various factors.^12,24,25,26^

Choi et al.^5^ used eyelid margin splitting and anterior lamellar repositioning in cases of lower eyelid marginal entropion. In this technique, the lid margin is split at the gray line, a subciliary incision is made and 6-0 vicryl sutures are placed to evert the anterior lamella. The procedure was successful in 27 of 30 patients (90%). In the other 3 patients, despite achieving correct eyelid position, irritation continued due to trichiatric lashes, requiring additional electrolysis and eyelash ablation. Tarsal fracture procedure was used in the current study. This technique allowed a high success rate in our small patient group during the relatively short follow-up period. Ideal eyelid position was achieved in all patients, none of whom had preoperative trichiatic lashes, without any further intervention.

Successful outcomes can be achieved in the treatment of marginal entropion with various surgical approaches. Among these approaches, the tarsal fracture technique has become a preferred option for several reasons including short surgical duration, not involving full-thickness incision, rapid healing, very low incidence of side effects, and little postoperative edema allowing for early restoration of cosmetic appearance.^11,27^ Reacher et al.^13^ reported that in cicatricial entropion cases, the success rate of tarsal fracture surgery was 80% in one study and 96.7% in another.^28^ Kersten et al.^12^ achieved a success rate of 85-93% using tarsal fracture technique for cicatricial entropion of the upper and lower eyelids unrelated to trachoma. Sodhi et al.^11^ performed tarsal fracture surgery on 92 patients with upper eyelid cicatricial entropion and reported a success rate of 28% (26/92). In their study, marginal entropion was not specifically addressed; their patient group included cases of mild cicatricial entropion such as marginal entropion as well as patients with eyelid closing defects (34/92), metaplastic lashes (28/92) and significant eyelid deformity (48/92). The surgery was successful for all cases with cicatricial entropion alone (n=20). This study demonstrates that tarsal fracture surgery is less successful when the cases’ specific characteristics are not considered. In our study we also achieved good eyelid position outcome in all patients with tarsal fracture surgery. However, to assess long-term success, studies with larger patient populations and longer duration are necessary.

Marginal entropion is a condition frequently seen in clinical practice, though it may be overlooked due to unfamiliarity. All patients presenting with eyelid malposition should undergo a detailed slit-lamp examination, and for patients evaluated as having trichiasis the eyelid margin should be carefully assessed. In cases diagnosed with marginal entropion, increased cicatrization can be prevented by avoiding repeated trichoablation, and with appropriate surgical intervention accompanied by active treatment of eyelid margin inflammation, patients can regain proper eyelid position.
